# Study of the error correction capability of multiple sequence alignment algorithm (MAFFT) in DNA storage

**DOI:** 10.1186/s12859-023-05237-9

**Published:** 2023-03-23

**Authors:** Ranze Xie, Xiangzhen Zan, Ling Chu, Yanqing Su, Peng Xu, Wenbin Liu

**Affiliations:** Institution of Computational Science and Technology, Guangzhou University, Guangzhou, 510006 China

**Keywords:** DNA storage, Multiple sequence alignment, Error correction, MAFFT

## Abstract

Synchronization (insertions–deletions) errors are still a major challenge for reliable information retrieval in DNA storage. Unlike traditional error correction codes (ECC) that add redundancy in the stored information, multiple sequence alignment (MSA) solves this problem by searching the conserved subsequences. In this paper, we conduct a comprehensive simulation study on the error correction capability of a typical MSA algorithm, MAFFT. Our results reveal that its capability exhibits a phase transition when there are around 20% errors. Below this critical value, increasing sequencing depth can eventually allow it to approach complete recovery. Otherwise, its performance plateaus at some poor levels. Given a reasonable sequencing depth (≤ 70), MSA could achieve complete recovery in the low error regime, and effectively correct 90% of the errors in the medium error regime. In addition, MSA is robust to imperfect clustering. It could also be combined with other means such as ECC, repeated markers, or any other code constraints. Furthermore, by selecting an appropriate sequencing depth, this strategy could achieve an optimal trade-off between cost and reading speed. MSA could be a competitive alternative for future DNA storage.

## Introduction

DNA is perhaps one of the oldest and best mediums for information storage. Vast amount of information can be stored in microscopic DNA molecules, lasting for thousands of years [1]. In the past decade, DNA storage has been an active research area because of its high information density and long-term stability. It is expected that progress in synthesis and sequencing technology will make DNA storage economically feasible in the foreseeable future [2, 3].

DNA storage can be modeled as a physical process consisting of five stages: DNA synthesis, polymerase chain reaction (PCR), sequence decay, sampling, and sequencing [4]. Due to the imperfectness of current synthetic biological technologies, these stages might lead to sequence loss and data corruption such as insertion-deletion-substitution (IDSs) errors. One unique feature of DNA storage is that a sequence usually corresponds to multiple reads in the reading process. Sequence loss in the sampling process can be addressed with increasing sequencing depth and using fountain coding [5–7]. However, it still remains a challenge to reliably recover data from the noisy reads.

To correct IDS errors, early works applied traditional error correcting codes such as Reed-Solomon Code [8–10], BCH code [11, 12], Hamming code [13], and LDPC code [14, 15]. They first selected sequences of the correct length, which were assumed to have fewer errors, and corrected some of their errors. This strategy of adding logical redundancy is only suitable in low-error situations (e.g. <  = 5%), because a large amount of sequences are excluded when the error rate is high. Researchers also attempted to directly solve the IDS errors by some heuristic algorithms, such as the hidden Markov model (HMM) [16], A* algorithm [17], and the graph searching algorithm [18]. Using information from multiple sequences, these algorithms may tolerate errors ranging from 5 to 15%, far higher than the ECC based methods. Recently, our group proposed a modulation-based storage architecture which could correct up to 40% errors while achieving a logical density of about 1 bits/nt [19].

Alternatively, given that DNA sequencing naturally generates multiple copies, another approach is to use multiple sequence alignment (MSA) algorithms, which have been successfully applied in bioinformatics to analyze sequence data. This has inspired Antkowiak et al. [20] and Yazdi et al. [21] to adopt a strategy which first inferred an approximate consensus sequence by MSA, and then corrected the remaining errors by other code constraints. Compared with other methods, MSA can solve the IDS errors without adding any logical redundancy or estimating any model parameter. This appealing feature motivates us to ask the following questions:

1. What is the limit of MSA in error correction? Or given sufficient sequencing depth, how many errors can it correct?
2. At a given error rate, how many sequences are required to reconstruct the original sequence?
3. When and how should MSA be combined with other means to achieve a good trade-off between efficiency and reliability?

We believe the answers to these questions will deepen our understanding of MSA’s error correction capability, and provide us with a basic principle to employ MSA effectively. In this paper, employing the most simple quaternary encoding and constraints, we use a typical MSA algorithm, MAFFT [22], to examine the error correction capability of MSA in DNA storage. Simulation results reveal that there is a phase transition of its recovering performance at about 20% errors. Below this critical value, MSA’s error correction performance could approach complete recovery with sufficient sequencing depth. Considering the storage cost and reading efficiency, our results indicate that MSA is the optimal choice in the low error regime. In the medium error regime, MSA aided by other means, such as repeated markers or ECC, may be a competitive solution. In addition, we find that MSA is robust to imperfect clustering. To the best of our knowledge, this is the first study focused on understanding the error correction capability of MSA.

## Relevant information

In bioinformatics, MSA has long been a well-studied problem. Given a set of strands, it tries to find a global alignment that minimizes the sum of pairwise distances. It is of supreme importance in biology and is mainly used for phylogenetic analysis, juxtaposing nucleotides that have been inherited from a common ancestral nucleotide in order to infer homology [23]. Aligning sequences this way has important applications in bioinformatics, and could be extremely useful in DNA storage, as the alignment itself could indicate the synchronization positions.

The basis of multiple sequence alignment is the Needleman–Wunsch dynamic programming [24]. However, its time complexity grows exponentially with respect to the number of sequences. Therefore, researchers mainly use heuristic methods such as progressive alignment, which although does not guarantee optimality, provides a sound estimate quickly. It does so by first computing all the pairwise distances to build a guide tree, then align the sequences based on the order in the guide tree.

Researchers have developed many heuristic MSA algorithms to align the sequences based on a guide tree. Some of them excel in speed, such as MUSCLE [25] and Kalign [26], while some are known for accuracy, such as SATe [27] and ProbCons [28]. In this paper, we use a MSA software, MAFFT (FFT-NS-2), as a proof of concept on MSA’s error correction capability because of its good accuracy and quasi-linear time for pairwise comparison [29].

Over the years, researchers have developed many MSA algorithms. Some excel in speed, such as MUSCLE [25] and Kalign [26], and some are known for accuracy, such as SATe [27] and ProbCons [28]. In this paper, we use the popular MSA software, MAFFT. By default, it chooses FFT-NS-2, the strategy optimized for speed, which is on par with the fastest MSA algorithms [29]. But it also provides strategies that are state-of-the-art in terms of alignment accuracy, such as L-INS-i [29], which is comparable to the most accurate algorithms.

MAFFT has good accuracy and takes only quasi-linear time for pairwise comparison. At first, it slides one sequence against another to find homologous, or highly similar segments, which only takes O(nlogn) time since it uses the Fast Fourier Transform (FFT). Then, it can use these identified segments to greatly reduce the area of the DP table, making it much faster than the original DP algorithm. Moreover, this pairwise sequence comparison can be extended to group-to-group alignment.

In short, due to the sequencing depth in this study, speed is essential. Hence, we use MAFFT (FFT-NS-2) as a proof of concept on MSA’s error correction capability.

## Results

MSA is a parameter-free error correction method whose performance is determined by the sequence copies used (or sequencing depth). We encode (00-A, 01-T, 10-G, 11-C) a text file named “The Grandmother” into 140 DNA sequences of 120 bases (8 bases for index and 112 bases for data). The error rate *p* ranges from 1 to 40% and sequencing depth is at most 4000. While there are specific patterns where error occurrence is more likely, for simplicity, we assume the location and type of base errors introduced in synthesis and sequencing are random. Hence, the IDS errors are distributed uniformly at random. For simulation experiments, we assume that all reads have been clustered correctly and we focus on the reconstruction problem of these clustered sequences using MAFFT. For each error rate and sequencing depth, the average results are obtained from 100 repeated experiments.

### The phase transition of the error correction capability

**Fig. 1:**
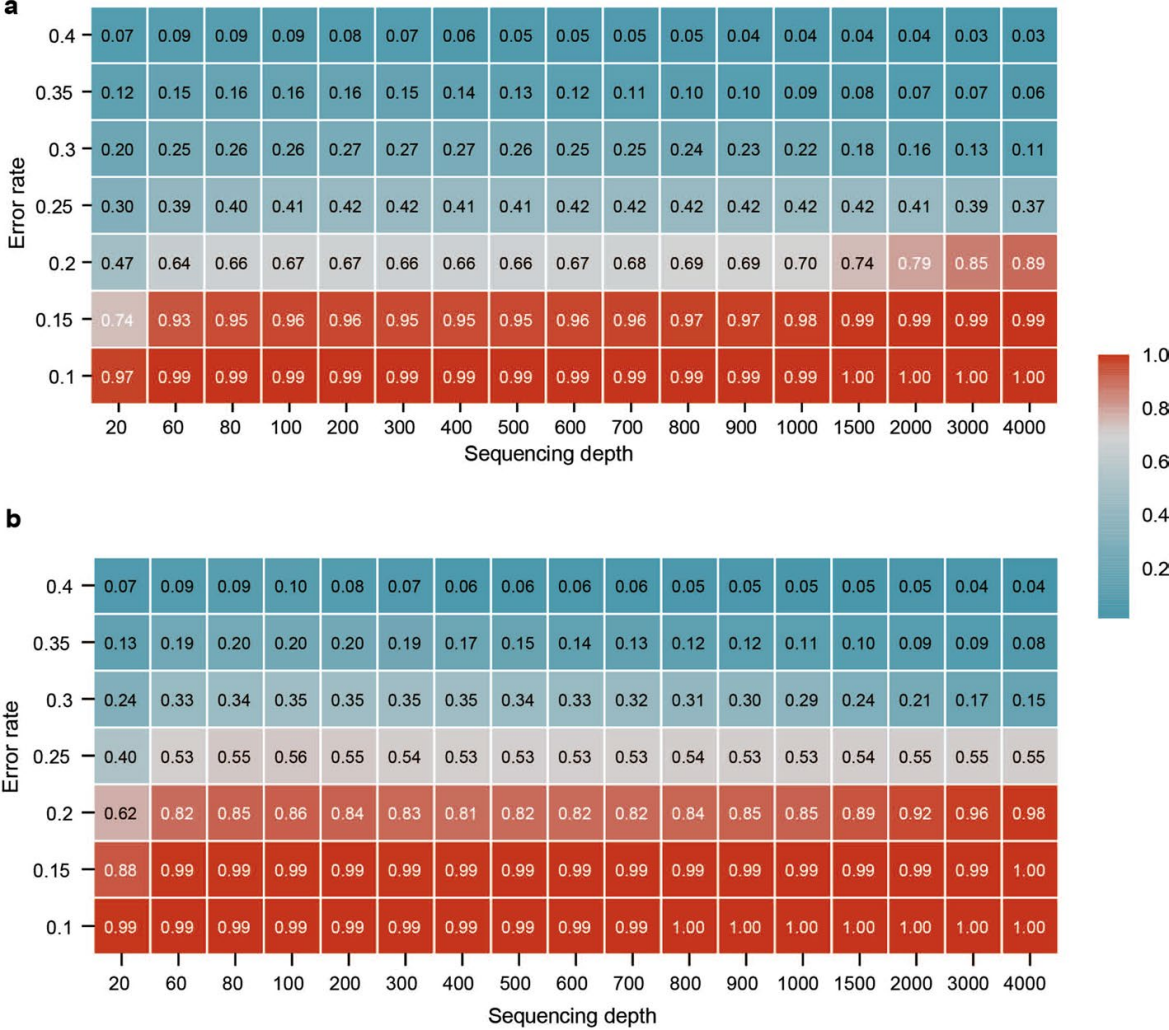
The phase transition of the error correction capability of MAFFT. **a** I:D:S ratio being 1:1:1. **b** I:D:S ratio being 1:1:2. The values in the grids denote the average recovery accuracy at each error rate and sequencing depth

Results indicate that MAFFT's error correction capability exhibits a phase transition. Figure 1 shows two phase transitions of the average recovery accuracy at two I:D:S ratios (1:1:1(a) and 1:1:2(b)), and the critical value is at about 20% error. When the error rate is less than the critical value, increasing sequencing depth may improve the average recovery rate (see the lower red region in Fig. 1). However, the needed sequencing depth dramatically increases as the error rate increases. On the contrary, the recovery accuracy drops steeply to even less than 50% in most cases when the error rate is larger than the critical value. And increasing sequencing depth could not improve the recovery accuracy (see the upper blue region in Fig. 1). This indicates that the synchronization errors have damaged the conserved structure among multiple sequences. Therefore, MSA could not capture any useful information from these sequences. These observations indicate that the limit of MAFFT is less than 20%.

In addition, the average recovery rate is related to the IDS ratio. Under the same conditions in Fig. 1a, b, we can see that the average recovery accuracy at I:D:S ratio 1:1:1 is lower than that of I:D:S ratio 1:1:2. This is coincident with our intuition that assuming a successful synthesis, the more insertions/deletions (indels) in the sequenced reads, the harder it is to solve the synchronization problem.

### The error correction capability

**Fig. 2:**
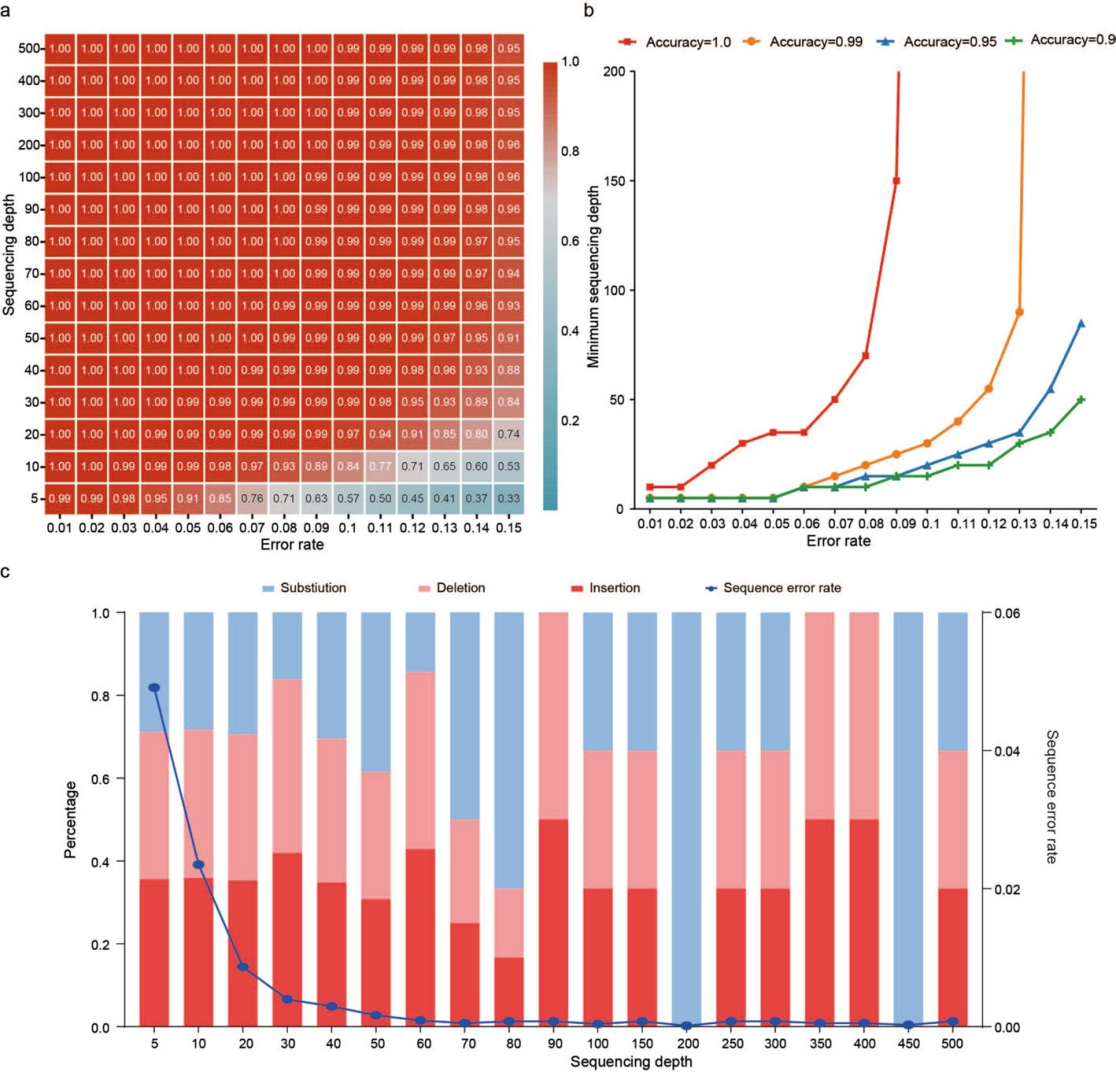
The error correction capability of MAFFT. **a** Recovery accuracy at each error rate and sequencing depth. **b** Minimum required sequencing depth for recovery accuracy 90% (green), 95% (blue), 99% (orange), and 100% (red). **c** The observed error correcting process of a sequence with 12% errors

Although MAFFT has the potential to completely recover data at error rates < 20%, it is highly impractical in terms of reading speed and cost to sequence thousands of reads for just one sequence. Because of the issue of sequence loss in the sampling process, sequencing depth between 50 and 100 seems to be a reasonable range for large-scale application [6, 8]. In this section, we further conduct a detailed study on the capability of MAFFT below error rate 15%. Figure 2a shows the average recovery accuracy at error rate 1%-15% and the corresponding sequencing depth 5–500. Generally, MAFFT could correct more than 95% of the errors at sequencing depth 100 when the error rate is below 15%.

Based on the error correction capability of MAFFT, we divide the error range into three regimes: the low (*p* ≤ 8%), medium (8% < *p* ≤ 15%) and high error regime (*p* > 15%). Here we focus on the low and medium regimes where it excels.

In the low error regime, MAFFT can completely recover the data. The red curve in Fig. 2b shows the required sequencing depth for 100% recovery accuracy. In this regime, it increases approximately linearly as the error rate increases. At *p* = 8%, MAFFT only needs 70 reads. Since we only used the simple quaternary encoding and constraints, this means that MAFFT can achieve complete recovery with reasonably little copies and minimal loss in logical density. Therefore, MSA may be a crucial part of the optimal solution to a reliable, fast, and low-cost DNA storage application in the low error regime.

In the medium error regime, MAFFT can correct more than 90% of the errors with a modest sequencing depth (≤ 100). The various curves in Fig. 2b show that, at a given error rate, the needed sequencing depth to reach higher accuracy increases dramatically. For example, at error rate 13%, to improve the accuracy from 90 to 95%, the needed sequence only increases from 30 to 35. However, this number increases from 35 to 60 in order to improve the accuracy from 95 to 99%. Figure 2c shows the error correcting process of a sequence with 12% errors. We observe that a significant portion of errors are still synchronization errors even with increased sequencing depth. This suggests that increasing sequencing depth beyond 60 seems to have negligible effect on correcting the few remaining indels. At this point, gaining higher accuracy at the expense of sequencing depth and reading speed may not be an efficient strategy. Therefore, it would be a wise alternative to combine MSA with other means, such as markers, ECC, or constraints on encoding. We could let MSA correct the vast majority of errors under a feasible sequencing depth, and then let other methods handle the rest. For example, we could let MSA handle 99% and 95% of the errors at *p* = 12% and 14%, where the needed sequencing depth is about 50. As most of these errors are solved by MSA, the added redundancy will be significantly reduced and thus still achieve a relatively high logical density approaching 2 bits/nt.

### Impact of clustering accuracy

**Fig. 3:**
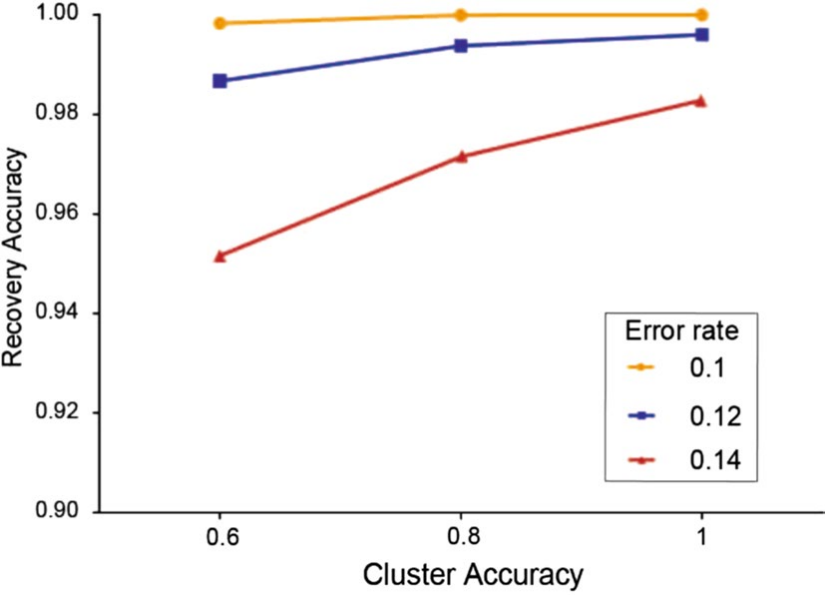
Recovery accuracy with respect to clustering accuracy at cluster size 100

The error correction capability of MAFFT is robust to imperfect clustering. In our simulation experiment, each cluster has 100 reads and we experiment with 0%, 20%, and 40% of these reads being noisy sequences, which correspond to cluster accuracy of 1, 0.8, and 0.6 in Fig. 3, respectively. Additionally, Fig. 3 shows the average recovery accuracy at 10%, 12%, and 14% error rates after repeating the experiment 500 times. Although the average recovery accuracy slightly decreased when we added noisy reads, they are all still greater than 0.95 even when the clustering accuracy is as low as 0.6. This may be explained by the post-alignment voting process’s ability to retain the conserved information and filter out most of the noises. That is, MSA could maximally utilize the reads’ information, even if they contain some noises.

### Decoding performance on a real dataset

To test the ability of MAFFT on real data, we download a real dataset (https://github.com/microsoft/clustered-nanopore-reads-dataset) published by Microsoft group [30]. It contains 10,000 DNA sequences of length 110 synthesized by Twist Bioscience and amplified using polymerase chain reaction. The final clustered file contains 269,709 noisy nanopore sequencing reads. In order to correct errors, they added some marker repeat (MR) codes in the original binary data and then translated them into DNA sequences according to a quaternary code (00-A, 01-T, 10-G, 11-C). It is estimated that the percentage of insertions, deletions, and substitutions are roughly about 1.7%, 2%, and 2.2%, respectively.

**Fig. 4:**
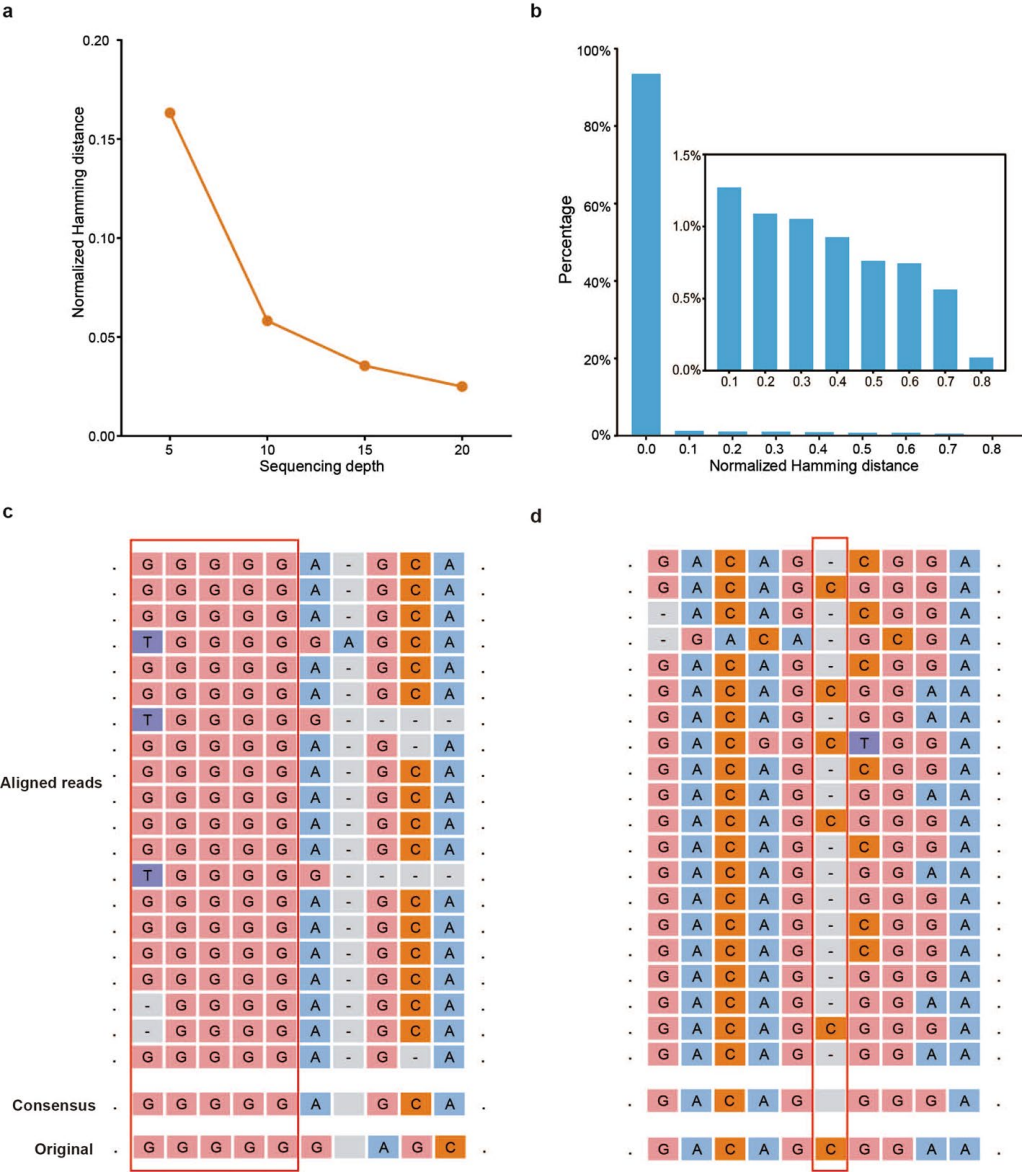
Recovery performance on a real nanopore sequencing dataset. **a** The average normalized Hamming distance at sequencing depth 5, 10, 15, and 20. **b** The distribution of normalized Hamming distance at sequencing depth 20. **c** An uncorrected example caused by base deletion in most reads. **d** An uncorrected example caused by misalignment

MAFFT could correct most of the errors in these sequences with only modest sequencing depth. Figure 4a shows the average normalized Hamming distance with different sequencing depths, and Fig. 4b shows the normalized Hamming distance distribution at sequencing depth 20. The leftmost bar in Fig. 4b indicates that MAFFT could completely recover about 92% of these sequences. Although the normalized Hamming distance may range from 0 to 0.8, most errors are caused by only a few indels. On the other hand, there are still some sequences which could not be completely recovered even with increased sequencing depth. Figure 4c, d shows two typical alignment errors. The first one is caused by a deletion of base ‘G’ in most reads. The second one is caused by misaligning some base C to the next position. Obviously, the former could not be solved because a base deletion in most reads would be irrecoverable by MSA, while the latter may be improved by increasing sequencing depth. In addition, we find that most of these errors usually occur when there are homopolymers, such as the example in Fig. 4c, which are highly prevalent in this dataset. It is well-known that homopolymers tend to introduce more IDS errors. And it seems that MSA has more trouble dealing with errors introduced by homopolymers, perhaps due to the nature of the algorithm. Therefore, constraining the length of homopolymers may reduce such errors for MSA.

## Discussions

It is well known that one of the main challenges in DNA storage is the synchronization problem. Results in previous sections have demonstrated that MSA is a potential method to deal with this issue. Previous works by Yazdi et al. [21] and Antkowiak et al. [20] also supported this point. The main advantage of MSA is that it can solve most of the IDS errors using the simplest quaternary encoding (minimal loss of logical density) while requiring only modest sequencing depths. The downside is that if a base was deleted in all the reads, which does have a non-negligible probability of occurring during synthesis, MSA alone has no way to recover it. Furthermore, the location and type of errors are not purely random, as they can be influenced by various error sources (GC content, homopolymers, reserved subsequences). This could lead to decreased error correction capability of MSA.

In order to achieve a reliable and large-scale storage application, we should make a suitable trade-off between cost and retrieval efficiency. The main cost in DNA storage comes from synthesis. Adding logical redundancy or coding constraints could improve error tolerance but would result in lower logical density and thus higher synthesis cost. On the other hand, retrieval efficiency is mainly determined by the average sequencing depth and decoding time complexity.

Although we only used MAFFT as a proof-of-concept of MSA’s error correction capability, we believe that the results we found are still largely applicable to other MSA algorithms. A more accurate MSA algorithm, such as SATe, while slower, would most likely require less sequencing depth to achieve the same recovery rate as MAFFT. It is also unlikely to have a higher practical error correction limit, since a sequencing depth in the thousands would be even more infeasible for these accurate but much slower algorithms. Therefore, we believe that MAFFT’s performance is a fair approximation of MSA algorithms’ overall error correction capability.

**Fig. 5:**
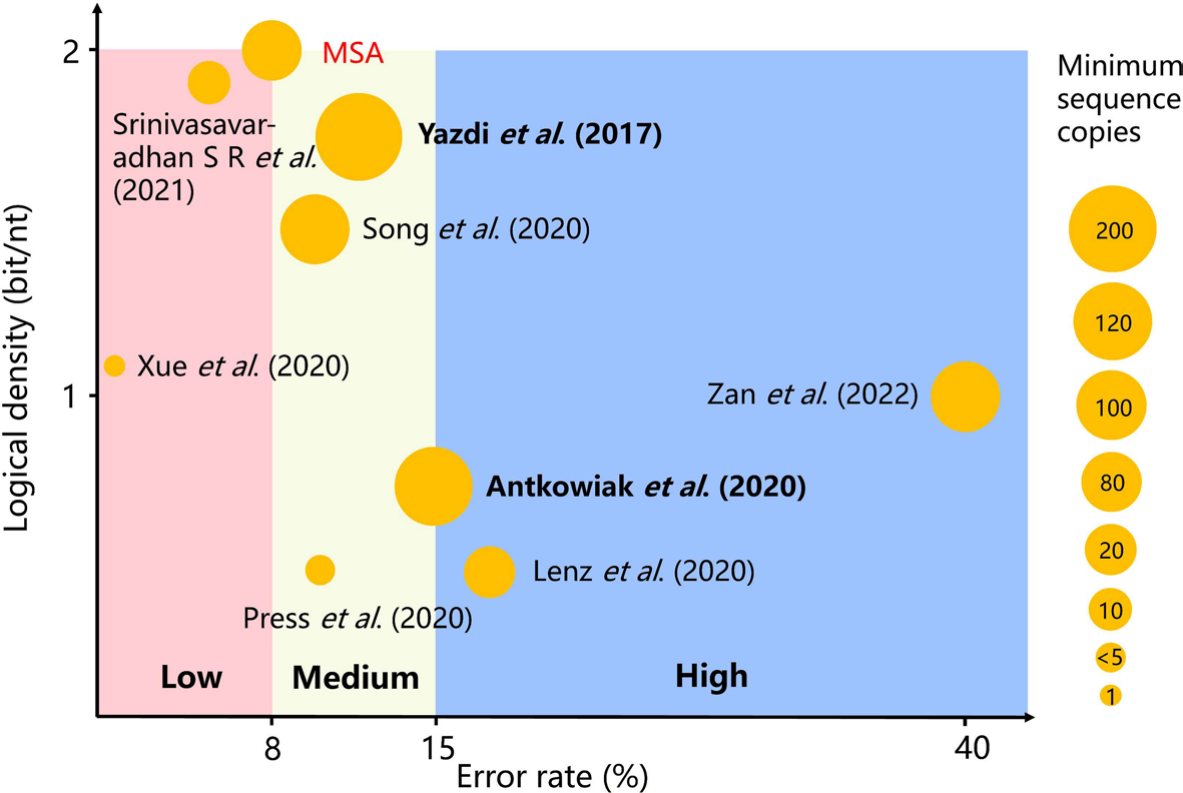
Landscape of the state-of-the-art methods in three error regimes

We plot the state-of-the-art methods in Fig. 5 according to their maximal tolerable error rates and logical densities. The average sequencing depth of these methods is indicated by the size of their orange circles. The error rate of the second-generation synthesis and sequencing technology is less than 5%, while that of the third-generation is about 10–15% [31, 32]. In practice, we could employ different error correction strategies according to the applied technology and application scenarios.

In the low error regime, MSA alongside the simplest encoding and constraints is perhaps the optimal choice: it achieves complete recovery with minimal loss in logical density while requiring only quasi-linear time complexity and modest sequencing depth. Therefore, MSA is suitable for applications where second-generation synthesis and sequencing technologies are used.

In the medium error regime, MSA aided with other means is a competitive solution. Compared with other methods in this regime, Yazdi et al. [21] has the most cost-efficient solution. Moreover, its logical density (1.75 bits/nt) is double that of Antkowiak et al.’s method (0.8 bits/nt) [20] with only about 2.5% reduction in error correction capability. From our results in Fig. 2b, the needed sequencing depth and logical redundancy of this method could be further optimized. This regime is suitable for ordinary storage applications where the third-generation synthesis and sequencing technologies could be used.

In the high error regime, the modulation-based method proposed by our group is perhaps the best solution today, as it could correct up to 40% errors. Both of its error correction capability and logical density are superior to the method proposed by Lenz et al. [16]. Since it uses a carrier strand to directly align the sequenced reads, MSA is thus not needed anymore. This regime is suitable for some extreme environments such as future low-cost high throughput technologies.

## Conclusion

MSA has some incomparable advantages over other proposed methods. First, it requires no redundancy which is crucial for high logical density. Second, its decoding process is quasi-linear which is beneficial for efficient information retrieval. Lastly, it can be combined with other means to provide a flexible architecture to design a MSA-dominated error correcting mechanism.

In this paper, we conduct a comprehensive study of the error correction capability of MSA for DNA storage. Our simulation results indicate that its error correction limit is about 20% errors. In the low error regime, MSA could accomplish complete recovery while retaining maximum logical density. In the medium error regime, a potential alternative is to apply MSA algorithm aided by other means to achieve a relatively high logical density. In sum, we believe that MSA would be a competitive paradigm in the low and medium error regimes for future DNA storage application.

## Data Availability

All data generated or analyzed during this study are included in this published article.
